# A large-scale survey of adverse events experienced in yoga classes

**DOI:** 10.1186/s13030-015-0037-1

**Published:** 2015-03-18

**Authors:** Tomoko Matsushita, Takakazu Oka

**Affiliations:** Faculty of Arts and Science, Kyushu University, Fukuoka, 816-8581 Japan; Department of Psychosomatic Medicine, Graduate School of Medical Sciences, Fukuoka, Japan

**Keywords:** Yoga, Adverse event, Risk factor, Stress, Large-scale survey

## Abstract

**Background:**

Yoga is a representative mind-body therapy of complementary and alternative medicine. In Japan, yoga is practiced widely to promote health, but yoga-associated adverse events have also been reported. To date, the frequencies and characteristics of yoga-related adverse events have not been elucidated. This study was conducted to elucidate the frequencies and characteristics of adverse events of yoga performed in classes and the risk factors of such events.

**Methods:**

The subjects were 2508 people taking yoga classes and 271 yoga therapists conducting the classes. A survey for yoga class attendees was performed on adverse events that occurred during a yoga class on the survey day. A survey for yoga therapists was performed on adverse events that the therapists had observed in their students to date. Adverse events were defined as “undesirable symptoms or responses that occurred during a yoga class”.

**Results:**

Among 2508 yoga class attendees, 1343 (53.5%) had chronic diseases and 1063 (42.3%) were receiving medication at hospitals. There were 687 class attendees (27.8%) who reported some type of undesirable symptoms after taking a yoga class. Musculoskeletal symptoms such as myalgia were the most common symptoms, involving 297 cases, followed by neurological symptoms and respiratory symptoms. Most adverse events (63.8%) were mild and did not interfere with class participation. The risk factors for adverse events were examined, and the odds ratios for adverse events were significantly higher in attendees with chronic disease, poor physical condition on the survey day, or a feeling that the class was physically and mentally stressful. In particular, the occurrence of severe adverse events that interfered with subsequent yoga practice was high among elderly participants (70 years or older) and those with chronic musculoskeletal diseases.

**Conclusions:**

The results of this large-scale survey demonstrated that approximately 30% of yoga class attendees had experienced some type of adverse event. Although the majority had mild symptoms, the survey results indicated that attendees with chronic diseases were more likely to experience adverse events associated with their disease. Therefore, special attention is necessary when yoga is introduced to patients with stress-related, chronic diseases.

## Introduction

Yoga is a representative mind-body therapy of complementary and alternative medicine. In Japan, yoga has been widely practiced to promote health, particularly among young women. Yoga has been reported to improve various stress-induced complaints of the mind and body, including anxiety, insomnia, and fatigue. The mechanism is gradually being elucidated regarding how yoga improves these symptoms. However, reports on yoga-associated adverse events have also been increasing. Glenn Black, a yoga teacher for almost 40 years, stated in a 2012 New York Times article that an increasing number of people have yoga-induced injuries and are in poor physical condition. These injuries include whiplash, muscle damage, and back strain. In addition, serious conditions such as stroke can also occur. Black has been warning people practicing yoga regarding these adverse effects of yoga [1].

Most reports on yoga-associated adverse events have been from randomized controlled clinical trials on the usefulness of yoga and case reports of individuals practicing yoga (for review, see [2]). Lower back pain and muscular pain are the most common symptoms in these reports [3,4]. While yoga has been suggested to relieve chronic neck pain and lower back pain [5], it has also been reported to adversely affect individuals by aggravation of pain [6-9]. Yoga has been indicated to cause musculoskeletal pain in healthy individuals [10], but such pain is mild in many cases. However, some musculoskeletal disorders are serious, such as bone fractures [11,12], tendon and ligament injuries [13,14], muscle strain [15], and myositis ossificans of the forearm [16]. Non-musculoskeletal disorders include ocular disorders such as keratectasia, central retinal vein occlusion, and progressive optic neuropathy in glaucoma patients [17-22], dyspnea and pneumothorax [23-25], and rectus sheath hematoma [26,27]. Rare adverse events are headache [28], sciatic nerve injury [29], hallucination [30], and dental erosion [31]. These adverse events were reported in articles only when they were unique or were seen in specific treatment settings. Only one web-based national survey in Australia investigated the yoga-related injury rate [32]. The results demonstrated that the incidence of yoga-related injuries was relatively low (21.3% of respondents reported some kind of yoga-related injury, and 4.6% sustained an injury in the previous 12 months). However, to date, no study has elucidated the frequency and causes of adverse events in regular yoga classes.

It is important to understand the characteristics, frequencies, and risk factors of yoga-associated adverse events before yoga becomes even more prevalent for stress reduction in healthy individuals and treatment of stress-related disorders. Our study involved a national survey that aimed (1) to elucidate the frequencies and characteristics of adverse events associated with yoga class, (2) to examine the risk factors of adverse events, and (3) to examine the condition of adverse events that yoga therapists observed in their students. In this study, adverse events are defined as “undesirable symptoms or responses that occurred during a yoga class”. Some of these results were reported previously in abstract form [33].

## Subjects and methods

### Subjects

The subjects were attendees of yoga classes taught by yoga therapists certified by the Japan Yoga Therapy Society and yoga therapists. The yoga classes were in 224 locations in 40 nationwide prefectures in Japan. There were 2508 class attendees (129 men and 2379 women) who responded to the survey. The mean age was 58.5 ± 12.6 years (mean ± standard deviation). There were 271 yoga therapists (13 men and 258 women) with a mean age of 54.1 ± 10.1 years. The attendees had taken yoga classes for a mean of 6.0 ± 5.56 years, and the yoga therapists had taught yoga for a mean of 10.7 ± 8.4 years.

### Methods

A self-administered questionnaire was conducted among attendees of a yoga class and yoga therapists. The attendees were asked about adverse events that had occurred during the class on the survey day. The yoga therapists were asked about adverse events that they had observed in their students to date.

The author of this study (Matsushita) explained the purpose and methods of this study to the yoga therapists in a seminar. A questionnaire for yoga therapists was given to the therapists, who provided written consent to participate in this survey. The yoga therapists explained the purpose and methods of the survey to yoga class attendees. A questionnaire for attendees was given to the attendees who provided written consent to participate in this survey. The survey period was between April and June 2013.

### Questionnaire

#### Questionnaire items for yoga class attendees

A list of physical and psychological symptoms was created based on the Cornell Medical Index (CMI). The class attendees were asked to check the symptoms that they had after taking the class and to report the symptoms using a free-response format. They were asked about their condition on the day of class: physical condition before participation on the day of the yoga class, effort in yoga class (level of overexertion), and physical and mental burden of yoga class (physical and mental strain). Other questionnaire items were on the presence or absence of chronic diseases and their details.

#### Questionnaire items for yoga therapists

The questionnaire items for yoga therapists were on adverse events that they had observed in their students to date. They were asked to rate the adverse events by severity (mild, moderate, and severe) and to indicate their frequencies. In addition, they were asked to write about the causes of the adverse events in a free-response format.

### Statistical analyses

Results are presented as mean ± standard deviation. To assess the risk factors for adverse events, we used the chi-square test and the multiple logistic regression test. Data were analyzed with SPSS ver.21 for Windows.

### Ethical considerations

This study was conducted with the approval of the ethics committee of the Institute of Health Science at Kyushu University. Informed consent was obtained from all subjects before the survey was conducted. Their written consent was obtained regarding the use of the questionnaire items. For subjects who were minors, informed consent was obtained from their parents.

## Results

### Frequency and characteristics of adverse events reported after a yoga class

**Figure 1:**
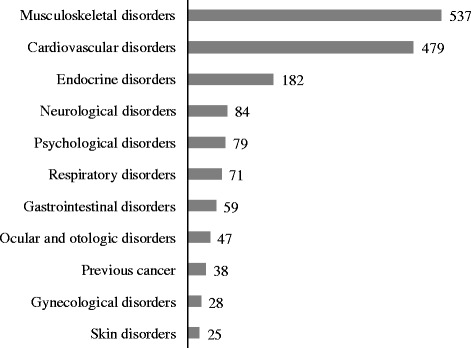
**Chronic diseases of yoga class attendees.**

Table 1 shows the demographic characteristics of yoga attendees who responded to the survey. Their ages ranged from 12 to 93 years and their mean age was 58.5 ± 12.6 years. They consisted of 129 men and 2379 women. There were 1343 attendees (53.5%) with chronic disease and 1063 attendees (42.3%) who were being treated at hospitals as outpatients. The most common chronic diseases were orthopedic disorders such as lower back pain and shoulder muscle stiffness in 537 attendees (21.4%), followed by cardiovascular disease such as hypertension in 479 attendees (19.0%), endocrine and metabolic diseases such as hyperlipidemia and diabetes mellitus in 182 attendees (7.2%), neurological diseases such as dysautonomia and headache in 84 attendees (3.3%), and psychiatric disorders such as depression and insomnia in 79 attendees (3.1%). Other chronic diseases included respiratory diseases, gastrointestinal disorders, and previous cancer (Figure 1).

**Table 1:** **Demographic characteristics of yoga class attendees**

**Age**	**Men**	**Women**	**Total**	**%**
10s	1	0	1	0.0%
20s	4	32	36	1.4%
30s	5	190	195	7.8%
40s	10	371	381	15.2%
50s	21	486	507	20.2%
60s	35	880	915	36.5%
70s	41	369	410	16.3%
80s	12	48	60	2.4%
90s	0	3	3	0.1%
Total	129	2379	2508	100.0%

#### Characteristics of adverse events

There were 687 attendees (27.4%) who reported some type of adverse event after a yoga class. Table 2 shows the specific symptoms and frequencies. The most common symptoms were of the musculoskeletal system, which were reported by 277 attendees (11.0%). These musculoskeletal symptoms were muscular pain in 132 attendees (5.3%), joint pain in 122 attendees (4.9%), and muscle cramp in 43 attendees (1.7%). The second most common symptoms were of the nervous system, which were reported by 237 attendees (9.4%). These neurological symptoms were dizziness in 101 attendees (4.0%), numbness in a certain body part in 47 attendees (1.9%), twitching in a certain body part in 41 attendees (1.6%), faintness in 33 attendees (1.3%), and heaviness of the head in 24 attendees (1.0%). The third most common symptoms were of the respiratory system, which were reported by 129 attendees (5.1%). These respiratory symptoms were coughing in 80 attendees (3.2%), nasal congestion in 31 attendees (1.2%) and runny nose in 27 attendees (1.1%). Symptoms affecting at least 1% of the attendees also included fatigue in 25 attendees (1.0%).

**Table 2:** **Symptoms and incidence of adverse events reported after yoga class**

	**Symptoms**	**n**	**Incidence**
Eyes and ears	Tinnitus	13	0.5%
	Blackout	7	0.3%
	Pruritus of the eye	4	0.2%
Respiratory system	Coughing	80	3.2%
	Congested nose	31	1.2%
	Runny nose	27	1.1%
	Sputum production	7	0.3%
Cardiovascular system	Breathlessness	22	0.9%
	Palpitation	13	0.5%
	Chest pain	1	0.0%
Gastrointestinal system	Gastric and abdominal pain	7	0.3%
	Nausea	6	0.2%
	Diarrhea	2	0.1%
Musculoskeletal system	Muscular pain	132	5.3%
	Joint pain	122	4.9%
	Foot and muscle cramp	43	1.7%
Skin	Flushing of the face	24	1.0%
	Pruritus of the skin	13	0.5%
	Excessive perspiration	10	0.4%
Neurological system	Dizziness	101	4.0%
	Numbness of a certain body part	47	1.9%
	Twitching in a certain body part	41	1.6%
	Faintness (dazed)	33	1.3%
	Heaviness of the head	24	1.0%
	Feeling of hotness and coldness	22	0.9%
	Headache	18	0.7%
Fatigue	Feeling of exhaustion	25	1.0%
	Feeling of illness	16	0.6%
Psychological symptoms	Tension	14	0.6%
	Shaking of the body	6	0.2%
	Anxiety	5	0.2%
	Recollection of bad experience	5	0.2%
	Gloominess	3	0.1%
	Feeling of wanting to cry	2	0.1%
	Irritation	2	0.1%
	Scary thoughts	1	0.0%
	Frightened feeling	1	0.0%
	Heightened emotion	1	0.0%
	Total	931	37.1%

#### Severity of adverse events

**Figure 2:**
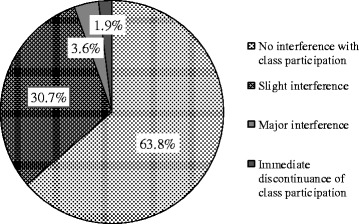
**Severity of adverse events occurring during yoga class.**

The severity of adverse events during a yoga class was rated on a 4-point scale: “no interference with subsequent class participation” “slight interference,” “major interference,” and “immediate discontinuance of class participation.” The attendees without interference from adverse events accounted for 63.8% of the attendees responding to questions on the severity of adverse events. The attendees with slight interference accounted for 30.7% of the attendees responding to such questions, the attendees with major interference accounted for 3.6%, and the attendees with immediate discontinuance accounted for 1.9% (Figure 2). In the attendees with major interference or immediate discontinuance, the adverse events included blackout, coughing, abdominal pain, muscular pain, joint pain, physical unsteadiness, and a feeling of illness. In this study, no one needed to call an ambulance. All of these symptoms improved gradually after stopping the activity and taking a rest.

### Risk factors for adverse events

#### Risk factors for adverse events

The risk factors for adverse events were examined by comparing the following items between the yoga class attendees with adverse events and those without: age, frequency of attending classes, condition on the day of class, and presence or absence of chronic disease. If factors showed a significant difference (p < 0.05) in univariate analysis using a chi-square test, their odds ratios and 95% confidence intervals (CI) were calculated (Table 3). Furthermore, multivariate analysis was performed using multiple logistic regression, and adjusted odds ratios were calculated (Table 4). The significant factors in univariate analysis were age (less than 40 years), condition on the day of class (severity of physical condition on that day, level of overexertion, physical strain, and mental strain), and chronic disease (respiratory disorders, musculoskeletal disorders, and neurological disorders). There was no difference by sex or frequency of attending classes. Multivariate analysis showed that factors considered significant by odds ratio analysis were age (less than 40 years) and condition on the day of class (severity of physical condition on that day, level of overexertion, physical strain, and mental strain), and chronic disease (only respiratory disorders and musculoskeletal disorders).

**Table 3:** **Risk factors for adverse events reported after yoga class**

**Factors**		**Chi-square value**	**P-value**	**Odds ratio (95% CI)**
Sex		2.67	0.125	
Age (less than 40 years)		14.65	0.000	1.73(1.30-2.30)
Frequency of attending classes		0.23	0.661	
Condition on the day of class				
	Severity of physical condition on that day	15.54	0.000	1.83(1.35-2.47)
	Level of overexertion	73.73	0.000	2.55(2.05-3.17)
	Physical strain	83.84	0.000	2.86(2.27-3.59)
	Mental strain	48.27	0.000	6.36(3.51-11.51)
Presence of chronic disease		20.08	0.000	1.52(1.26-1.82)
	Ocular and otologic disorders	0.31	0.625	
	Respiratory disorders	7.22	0.011	1.90(1.18-3.07)
	Cardiovascular disorders	0.03	0.909	
	Gastrointestinal disorders	0.61	0.462	
	Musculoskeletal disorders	16.72	0.000	1.54(1.25-1.89)
	Skin disorders	3.35	0.075	
	Neurological disorders	4.69	0.035	1.63(1.04-2.57)
	Psychological disorders	0.03	0.897	
	Endocrine disorders	1.53	0.228	
	Gynecological disorders	0.28	0.671	
	Previous cancer	0.13	0.852

**Table 4:** **Multivariate analysis of factors associated with adverse events reported after yoga class**

**Factors**	**Odds ratio**	**95% Confidence interval**	**P-value**
Age (less than 40 years)	1.95	1.44-2.63	<0.001
Condition on the day of class			
Severity of physical condition on that day	1.58	1.14-2.18	0.006
Level of overexertion	1.82	1.41-2.34	<0.001
Physical strain	1.89	1.45-2.47	<0.001
Mental strain	3.93	2.11-7.33	<0.001
Presence of chronic disease	1.29	1.03-1.62	0.030
Respiratory disorders	1.78	1.06-2.97	0.028
Musculoskeletal disorders	1.30	1.01-1.67	0.042
Neurological disorders	1.39	0.86-2.26	0.180

The risk factors were examined for adverse events that interfered with subsequent class participation by comparing the attendees with such adverse events (slight interference, major interference, and discontinuance of participation) and those with adverse events that did not interfere with participation. The significant factors in univariate analysis were age of 70 years or older (OR = 2.41,95% CI: 1.27-4.61, p < 0.01), physical condition on that day (OR = 2.11, 95% CI: 1.11-4.01, p < 0.05), and chronic musculoskeletal disorders (OR = 1.74, 95% CI: 1.05-2.86, p < 0.05). Multivariate analysis showed that the only factors considered significant by adjusted odds ratio analysis were age 70 years or older (OR = 2.25, 95% CI: 1.10-4.59, p < 0.05) and physical condition on that day (OR = 1.99, 95% CI: 1.02-3.89, p < 0.05).

#### Relationship between chronic disease and risk of adverse events

The relationship between the systems affected by chronic diseases and the risk of adverse events related to the affected systems was examined. The odds ratio and 95% CI were calculated for factors with p < 0.05 in univariate analysis using a chi-square test. The results showed that the risk of musculoskeletal system adverse events was higher in the attendees with chronic musculoskeletal disorders compared with those without (OR = 2.25, 95% CI: 1.10-4.59, p < 0.05). Of the musculoskeletal adverse events, muscular pain (OR = 2.25, 95% CI: 1.10-4.59, p < 0.05) and joint pain (OR = 2.25, 95% CI: 1.10-4.59, p < 0.05) showed significant association with chronic musculoskeletal disorder. The attendees with chronic respiratory disorders had a higher incidence of respiratory adverse events (OR = 4.77, 95% CI: 2.58-8.81, p < 0.001). Of the respiratory adverse events, the following events showed significant association with chronic respiratory disorders: coughing (OR = 2.92, 95% CI: 1.22-6.96, p < 0.05), congested nose (OR = 6.77, 95% CI: 2.52-18.17, p < 0.001), and runny nose (OR = 10.75, 95% CI: 4.18-27.64, p < 0.001). The attendees with previous cancer had a high risk of respiratory adverse events (OR = 2.99, 95% CI: 1.14-7.82, p < 0.05). Of the respiratory events, coughing (OR = 3.70, 95% CI: 1.28-10.68, p < 0.05) showed a significant association with previous cancer. The attendees with chronic neurological disorders showed a higher incidence of ocular and otologic adverse events (OR = 6.09, 95% CI: 2.02-18.32, p < 0.01) and gastrointestinal adverse events (OR = 5.37, 95% CI: 1.53-18.78, p < 0.01) compared with the attendees without them. Of these adverse events, tinnitus (OR = 14.65, 95% CI: 4.32-49.66, p < 0.001) and gastric and abdominal pain (OR = 11.45, 95% CI: 2.19-59.89, p < 0.01) showed significant association with chronic neurological disorders.

### Adverse events in students observed by yoga therapists

In this study, 271 yoga therapists were asked about adverse events that they had observed to date in their students during yoga classes. They divided the adverse events into three categories. (1) Mild events were temporary and the students were able to continue class participation. (2) Moderate events required the students to discontinue class participation, to take a wait-and-see approach, and to rest. (3) Severe events required the students to discontinue class participation and to be examined or treated by a physician. In addition, they were asked to use a free-response format to describe the characteristics of the moderate and severe adverse events.

#### Severity of adverse events

There were 229 yoga therapists (84.5% of the therapists who responded) who had observed to date mild adverse events during yoga classes, 81 therapists (30.0%) had observed moderate adverse events and 22 therapists (8.1%) had observed severe events.

#### Characteristics of moderate and severe adverse events

The therapists reported 93 cases of moderate or severe adverse events (Table 5). Eight cases required emergency transport. These events included post-class subarachnoid hemorrhage, subluxation of the hip joint, backward fall, sudden attack of abdominal pain, dizziness, arrhythmia, hyperventilation, and inability to move due to illness and increased anxiety. There were 14 cases for which the students were examined at medical institutions. These adverse events included contusion and bone fracture from a fall due to loss of balance, fall due to illness, illness due to elevation of blood pressure, fall due to physical unsteadiness, severe pain of the hip joint, knee joint and abdomen, pain due to meniscal injury and Achilles tendon rupture, hyperventilation, dizziness, and palpitation. There were 46 other cases in which the attendees discontinued class participation and rested due to symptoms such as dizziness, physical unsteadiness, illness, muscular pain, fall, and hyperventilation. There were nine cases in which instructions were given to control breathing, and three cases in which massage was given.

**Table 5:** **Moderate and severe adverse events observed by yoga therapists**

	**Symptoms**	**n**
Eyes and ears	Blackout	1
Respiratory system	Difficult breathing and hyperventilation	7
	Rapid breathing	1
Cardiovascular system	Breathlessness	2
	Palpitation	2
	Heart attack	2
	Chest tightness	1
	Arrhythmia	1
Gastrointestinal system	Nausea	4
	Abdominal pain	3
Musculoskeletal system	Muscular pain	11
	Joint pain	3
	Foot and muscle cramp	3
	Bone fracture	2
	Achilles tendon rupture	1
	Meniscal injury	1
	Subluxation of the hip joint,	1
Skin	Perspiration, cold sweat, and stress-induced perspiration	4
	Pruritus of the skin	1
Neurological system	Dizziness and physical unsteadiness	20
	Fall	9
	Headache	2
	Dazed	1
	Cold limbs	1
	Faintness	1
	Shaking of the body	1
	Flushing of the body	1
	Numbness of the body	1
Fatigue	Feeling of unwellness	18
Psychological symptoms	Recollection of bad experience	4
	Heightened emotion	2
	Confusion	2
	Tension	1
Other	Subarachnoid hemorrhage	1
	Pain of surgical scar	1
	Other	3

#### Possible causes of adverse events

**Figure 3:**
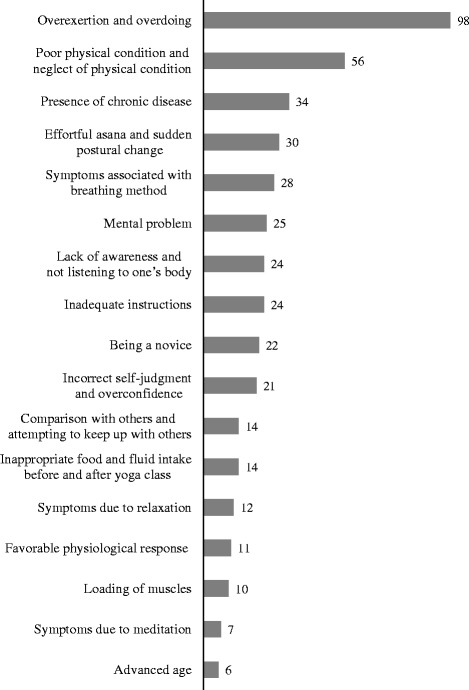
**Causes of adverse events in the opinion of yoga therapists (ranging from mild to severe events).**

Figure 3 shows the causes of the mild to severe adverse events in the opinion of the yoga therapists teaching the class. Many causes were associated with the students such as “overexertion and overdoing” and “poor physical condition and neglect of physical condition.” The specific causes included effortful breathing method causing coughing, overloading causing pain and cramp of the limbs, psychological problems causing hyperventilation, and meditation causing recollection of bad experiences. Other causes were attributed to the yoga therapists: “inadequate instructions” (i.e., the therapists did not notice because they were not aware of the students’ chronic diseases or health conditions) and inadequate observation and verbal communication. In addition, some yoga therapists thought the adverse events were transient symptoms caused by improved blood flow and muscle relaxation that result in alleviation of symptoms in the long term. Such alleviating events were “favorable physiological response” and “symptoms due to relaxation” and included pruritus and temporary pain.

## Discussion

This study examined the characteristics and frequencies of adverse events occurring during yoga classes in 2508 class attendees. It also examined the characteristics and frequencies of adverse events observed to date by 271 yoga therapists. Our survey showed that the class attendees with chronic disease accounted for 54% of the class attendees and the attendees who were hospital outpatients accounted for 42% of the attendees. These results show that, in Japan, people who take yoga classes are not necessarily healthy individuals and that many patients receiving treatment for their disease take classes to improve health. These chronic diseases were wide ranging and included orthopedic conditions, such as lower back pain and shoulder muscle stiffness, cardiovascular diseases, endocrine diseases, neurological diseases, and psychological disorders. Clinical effects and indications of yoga have not necessarily been established for these diseases. Therefore, unexpected adverse events can occur, and caution is required when yoga therapy is performed for patients with these diseases.

The results of this study demonstrated that 27% of the yoga class attendees experienced some type of adverse event during class. The most common adverse events were of the musculoskeletal system such as muscular pain, joint pain, and muscle cramp, and 11% of the attendees complained of these symptoms. In previous studies, the most commonly reported adverse events were also musculoskeletal symptoms [3,4,6-9]. In our study, 1% or more of the clients reported adverse events that are infrequently discussed in previous studies. These adverse events included neurological symptoms, such as dizziness and numbness, and respiratory symptoms, such as coughing. In our study, the adverse events reported by the class attendees were mostly mild and did not interfere with subsequent class participation.

The following factors were found to increase the risk of adverse events: age (less than 40 years), condition on the day of class (poor physical condition, overexertion, a sense of physical strain, and a sense of mental strain), and chronic diseases (presence of respiratory and musculoskeletal disorders). Mental strain was the factor with the highest odds ratio, and the class attendees with mental strain had an odds ratio of approximately 4 times higher risk for adverse event. Thus, a sense of mental strain from yoga class may be a good indicator of a risk of adverse events. The risk factors that interfered with subsequent class participation were age of 70 years or older, physical condition on that day, and presence of chronic musculoskeletal disorder. Although the occurrence of adverse events was high among individuals less than 40 years, adverse events were more severe in elderly individuals 70 years or older. Previous studies stated that the elderly were more likely to be affected by sudden postural and blood pressure changes, that caution is required for specific poses in osteoporotic individuals, and that considerations for safety are needed, such as automated external defibrillators in yoga studios [34,35]. Elderly individuals are likely unable to handle as much physical load as young individuals and are slower to recover from adverse events. Therefore, yoga should be performed carefully based on the individual’s disease and physical condition on that day. Special attention is necessary when elderly people practice yoga.

As to the relationship between diseases and adverse events, individuals with musculoskeletal diseases had significantly higher occurrences of adverse events, such as muscular pain and joint pain. Individuals with respiratory diseases had significantly higher occurrences of the adverse events of coughing, nasal congestion, and runny nose. These results suggest that patients with specific diseases may develop or exacerbate their disease-related symptoms by practicing yoga. Although the reason is uncertain, individuals with a history of cancer had significantly higher occurrences of the adverse event of coughing. Kaley-Isley et al. showed that the risk of adverse event was high in individuals with diseases such as intervertebral disk disease, extremely high or low blood pressure, glaucoma, retinal detachment, and atherosclerosis [36]. DiStasio stated that patients with symptomatic anemia, orthostatic hypotension, and lightheadedness should avoid prolonged standing poses, that cancer patients with fever and systemic infection should avoid vigorous yoga poses [37], and that patients with signs of osteoarthritis are not recommended to do yoga [5]. Since individuals with various chronic diseases practice yoga, it is important for these individuals to let the yoga therapists know in advance about their diseases [36,38]. Certain yoga poses or the practice of yoga itself might be prohibited or not recommended for individuals with certain conditions or diseases.

The survey for yoga therapists showed that 84.5% of the yoga therapists had observed mild adverse events in their students. The adverse events that required emergency transport included subarachnoid hemorrhage, subluxation of the hip joint, backward fall, attack of abdominal pain, and inability to move due to illness and increased anxiety. The details are unknown regarding the causal relationship between these events and yoga, but yoga therapists need to be able to respond to such events. Previous studies have reported on individuals with adverse events who required examination at medical institutions. These events included bone fracture, Achilles tendon rupture, and dyspnea [11-14,23-25]. The frequencies of these events are low, but it is necessary to devise measures in case they occur. Although severe ocular disorders were reported in other previous studies [17-22], such disorders were not reported in our survey. In our study, when the yoga therapists were asked about the causes of adverse events, they provided many factors associated with yoga class attendees: condition of attendees in class (such as overexertion, overdoing, and neglect of physical condition), presence of disease, and age. These therapists’ impressions were consistent with the findings based on the analysis of this study. Some instructors responded that they could not provide sufficient instructions to their students, partly because they did not know their students’ diseases and health conditions. Other instructors mentioned that the students did not know how much effort to put into yoga classes.

Healthcare providers and yoga therapists need to share medical information, especially the potential risks of the attendees, and to be aware of the possible adverse events that could occur, depending on the patient’s disease, the yoga pose, and other contents of the yoga class. It is also desirable for healthcare providers and yoga therapists to educate the yoga class attendees, including providing adverse event-related information in advance. When individuals with chronic diseases or risk factors for adverse events want to practice yoga, they should practice carefully, under the guidance of qualified yoga therapists. Furthermore, when severe adverse events do happen in the class, the information should be shared among all yoga therapists, and a follow-up system should be established to avoid such events in the future.

This study had several limitations. First, a very high percentage of the subjects were women who were 40 years or older. The mean age of the yoga class attendees (58.5 years) in this study might be higher than that in previous studies, i.e., 41.4 years in a study in Australia [32] and 46.7 years in a study in England [39]. However, this number may reflect typical yoga students in Japan, because the survey samples were obtained not from special settings, but from typical sports gyms and community centers throughout Japan. Second, the yoga class attendees filled out the questionnaire immediately after class. Thus, the data did not include adverse events occurring a few days after class. Third, our study did not examine physiological parameters, such as blood pressure, or laboratory test results, such as blood glucose levels. Therefore, adverse events associated with abnormalities of these parameters were not examined in this study. Future studies should be conducted to survey more young individuals and men, to examine longer term effects involving follow-up surveys of a few days to months after yoga class, and to examine laboratory test results and physiological parameters.

## Conclusions

The results of our large-scale survey demonstrated that approximately 30% of yoga class attendees had experienced some type of adverse event. Although most adverse events were mild, some individuals experienced severe events, which caused them to discontinue the class. This study also showed that the following factors can increase the risk of adverse events: age, presence of chronic disease, and condition of attendees on the day of class, such as poor physical condition on that day and overexertion. Therefore, special attention is necessary when yoga is introduced to patients with stress-related, chronic diseases.
